# Influence of pathogen and focus of infection on procalcitonin values in sepsis: are there additional confounding factors?

**DOI:** 10.1186/s13054-019-2499-1

**Published:** 2019-06-13

**Authors:** Patrick M. Honore, David De Bels, Rachid Attou, Sebastien Redant, Andrea Gallerani, Kianoush Kashani

**Affiliations:** 1 ICU Department, Centre Hospitalier Universitaire Brugmann-Brugmann University Hospital, Place Van Gehuchtenplein,4, 1020 Brussels, Belgium; 2 Division of Nephrology and Hypertension, Division of Pulmonary and Critical Care Medicine, Mayo Clinic, Rochester, USA

We read the study by Thomas-Rüddel et al. with great interest [1]. Authors showed serum procalcitonin (PCT) concentrations were higher in patients with Gram-negative bacteremia (26 ng/ml) than in those with Gram-positive bacteremia (7.1 ng/ml) or candidemia (*P* < .0001) [1]. They outlined some potential factors that could have impacted the PCT measurement outside to the type, location, and severity of the infection. In addition to their findings, it is essential to highlight that in patients with positive blood culture, septic shock is very common. Acute kidney injury (AKI) is prevalent among patients with sepsis, and a considerable proportion of patients with sepsis-associated AKI (SA-AKI) require renal replacement therapy (RRT) [2]. As PCT has an approximate molecular weight of 14.5 kDa [3], the contemporary continuous RRT membranes are able to remove it (CRRT cutoff is about 35 kDa) [4]. Also, using newer high adsorptive membranes (HAM) would make PCT removal even more prominent [4]. Accordingly, if in the study by Thomas-Rüddel et al., there was any imbalance between the uses of CRRT between the two groups, it could critically impact the observed results. We, therefore, suggest including the use of CRRT in the prediction model. In addition, the design of future studies to assess the performance of PCT among septic patients who are on CRRT seems to be necessary [5].

## Data Availability

Not applicable.

## Abbreviations

AKI: Acute kidney injury
CRRT: Continuous renal replacement therapy
HAM: Highly adsorptive membranes
PCT: Procalcitonin
RRT: Renal replacement therapy
SA-AKI: Sepsis-associated AKI

## References

[1] Thomas-Rüddel DO, Poidinger B, Kott M, Weiss M, Reinhart K, Bloos F; MEDUSA study group. Influence of pathogen and focus of infection on procalcitonin values in sepsis patients with bacteremia or candidemia. Crit Care 2018;22(1):128. doi: 10.1186/s13054-018-2050-9. 10.1186/s13054-018-2050-9 PMC5949148 29753321

[2] Peters E, Antonelli M, Wittebole X, Nanchal R, François B, Sakr Y (2018). A worldwide multicentre evaluation of the influence of deterioration or improvement of acute kidney injury on clinical outcome in critically ill patients with and without sepsis at ICU admission: results from The Intensive Care Over Nations audit. Crit Care.

[3] Level C, Chauveau P, Guisset O, Cazin MC, Lasseur C, Gabinsky C (2003). Mass transfer, clearance, and plasma concentration of procalcitonin during continuous venovenous hemofiltration in patients with septic shock and acute oliguric renal failure. Crit Care.

[4] Honoré PM, De Bels D, Spapen HD (2018). An update on membranes and cartridges for extracorporeal blood purification in sepsis and septic shock. Curr Opin Crit Care.

[5] Honoré PM, Jacobs R, De Waele E, Van Gorp V, Spapen HD (2014). Evaluating sepsis during continuous dialysis: are biomarkers still valid?. Blood Purif.

